# Loss of ISWI ATPase SMARCA5 (SNF2H) in Acute Myeloid Leukemia Cells Inhibits Proliferation and Chromatid Cohesion

**DOI:** 10.3390/ijms21062073

**Published:** 2020-03-18

**Authors:** Tomas Zikmund, Helena Paszekova, Juraj Kokavec, Paul Kerbs, Shefali Thakur, Tereza Turkova, Petra Tauchmanova, Philipp A. Greif, Tomas Stopka

**Affiliations:** 1 Biocev, 1st Medical Faculty, Charles University, 25250 Vestec, Czech Republic; tomzikmund@gmail.com (T.Z.); paszekova.helena@gmail.com (H.P.); juraj.kokavec@gmail.com (J.K.); shefalithakur.st@gmail.com (S.T.); tereza.turkova@volny.cz (T.T.); petra-tauchmanova@seznam.cz (P.T.); 2 Department of Medicine III, University Hospital, LMU Munich, D-80539 Munich, Germany; paul.kerbs@med.uni-muenchen.de (P.K.); pgreif@med.uni-muenchen.de (P.A.G.); 3 German Cancer Consortium (DKTK), partner site Munich, D-80336 Munich, Germany; 4 German Cancer Research Center (DKFZ), D-69120 Heidelberg, Germany

**Keywords:** SMARCA5, SNF2H, AML, leukemia, CRISPR, therapeutic target

## Abstract

ISWI chromatin remodeling ATPase SMARCA5 (SNF2H) is a well-known factor for its role in regulation of DNA access via nucleosome sliding and assembly. SMARCA5 transcriptionally inhibits the myeloid master regulator PU.1. Upregulation of SMARCA5 was previously observed in CD34+ hematopoietic progenitors of acute myeloid leukemia (AML) patients. Since high levels of SMARCA5 are necessary for intensive cell proliferation and cell cycle progression of developing hematopoietic stem and progenitor cells in mice, we reasoned that removal of SMARCA5 enzymatic activity could affect the cycling or undifferentiated state of leukemic progenitor-like clones. Indeed, we observed that CRISPR/cas9-mediated *SMARCA5* knockout in AML cell lines (S5KO) inhibited the cell cycle progression. We also observed that the *SMARCA5* deletion induced karyorrhexis and nuclear budding as well as increased the ploidy, indicating its role in mitotic division of AML cells. The cytogenetic analysis of S5KO cells revealed the premature chromatid separation. We conclude that deleting SMARCA5 in AML blocks leukemic proliferation and chromatid cohesion.

## 1. Introduction

Acute myeloid leukemia (AML) is a malignant hematopoietic disease derived from myeloid-primed stem cells resulting in accumulation of myeloid blasts. AML patients have a poor prognosis and the only known efficient therapy is bone marrow transplantation combined with chemotherapy. Next-generation sequencing revealed that despite similar cytology and cellular features, the mutational profile of AML clones can be very heterogenic. Leukemogenesis involves multiple types of genomic alterations from single nucleotide variants to large chromosomal abnormalities (involving deletions, translocations, or chromosomal gains and losses). Targets of mutagenesis are often genes encoding regulators of gene transcription (e.g., *RUNX1*, *CEBPA*, *GATA2*), DNA methylation (e.g., *DNMT3A*, *IDH1*, *IDH2*), and genome organization (e.g., *CTCF*, *RAD21*, *SMC3*). 

Immature cells during tissue development require ATP-dependent chromatin remodeling activities to ensure accession of regulatory proteins to DNA in order to control replication, transcription, or DNA repair. Activities that facilitate nucleosome spacing and assembly during tissue development are provided mainly by evolutionary conserved Swi2/Snf2 family helicases. Smarca5 (also known as Snf2h) belongs to important enzymes of the Swi2/Snf2 family with remodeling activity that is required for successful hematopoietic development in mammals [1,2,3]. In mouse, Smarca5 represents the catalytic subunit of ISWI remodeling complexes that is indispensable for developing embryo and later for fetal hematopoiesis [1,2]. Interestingly, Smarca5 loss was accompanied by upregulation of p53 and of its transcriptional targets that are usually linked to the induction of apoptosis in response to DNA damage (e.g., p21/Cdkn1a, Noxa/Pmaip1, and Bax) [1]. Our work and the work of others suggested that Smarca5 not only facilitates proliferation-associated events but also helps to activate transcriptional programs of particular developmental stages to set proper expression identity of immature cells [4,5]. Additional evidence implicated that Smarca5 regulates global gene expression programs and function of many human gene regulatory elements by cooperating with CTCF [6,7,8].

Smarca5 represents an integral part of heterodimeric ISWI complexes that contain usually a bromodomain-containing protein (BAZ1A, BAZ1B, BAZ2A, BAZ2B). ISWI complexes were originally identified in *Drosophila* but later they were discovered also in humans, namely, NURF (ATPase motor of the nucleosome remodeling factor), ACF (ATP-utilizing chromatin assembly and remodeling factor), and CHRAC (chromatin assembly complex). Later, additional human complexes were found, such as RSF, NoRC, WICH, CERF, and finally, SNF2H-cohesin [9]. Most ISWI complexes are involved in regulating cell cycle progression albeit via different mechanisms. While many ISWI complexes regulate transcription by nucleosome sliding mechanism utilizing either RNA-Polymerase 1 (RNAP1) (NoRC, B-WICH) or RNAP2 (ACF, NURF, CERF, WINAC), other complexes are linked to replication/repair (CHRAC, WICH) or chromatid cohesion (SNF2H-cohesin) [10]. It appears that SMARCA5 plays an indispensable part in the ISWI complexes (albeit it can remodel chromatin alone in acellular systems); however, in certain situations, it may be replaced within ISWI complexes by its close homologue SMARCA1 (SNF2L) as shown in rather differentiated cells of the cerebellum [4].

Currently, over 20% of all malignancies carry mutations in one of the subunits of chromatin remodeling complexes of the SWI/SNF family (see [11,12]). These mutations often decrease protein stability and cause loss of the particular subunit, which leads to the assembly of incomplete remodeling complexes with different functions in vivo and altered capability to precisely regulate gene expression [13]. In the case of the ISWI subfamily, the mutations of various ISWI subunits identified in oncologic diseases have still yet unknown impact on tumorigenesis. In solid tumors the overexpression of SMARCA5 [14,15,16,17,18] has been associated with disease aggressiveness, chemoresistance and proliferation activity [7]. SMARCA5 expression was found dysregulated in many human malignant tumors, such as aggressive gastric cancer, breast cancer, or prostate cancer. In addition, the *SMARCA5* gene is a target of cancer-associating miRNA regulation [14,15,16,17,18]. SMARCA5 overexpression has been also observed in AML CD34+ progenitors [7,19]. SMARCA5, through the interaction with CTCF in leukemic cells, actively inhibits expression of the *SPI1/PU.1* gene [7] that represents key hematopoietic transcription factor and dose-dependent leukemia suppressor [20]. Additional work utilizing the CRISPR/Cas9 genome editing in vitro revealed that among hematopoietic cancer cell lines, those derived from AML patients were the most SMARCA5 dependent [21]. We herein studied the consequences of *SMARCA5* deletion in AML cells and showed that SMARCA5 targeting affected proliferation and resulted in chromosomal aberrations and polyploidy pointing to the role of SMARCA5 in mitotic division. We believe that delineating the effects of *SMARCA5* targeting might pave the way for new approaches in the therapy of AML.

## 2. Results

### 2.1. SMARCA5 Overexpression Marks the Hyperproliferation and Cytogenetically Abnormal AML Patients

**Figure 1:**
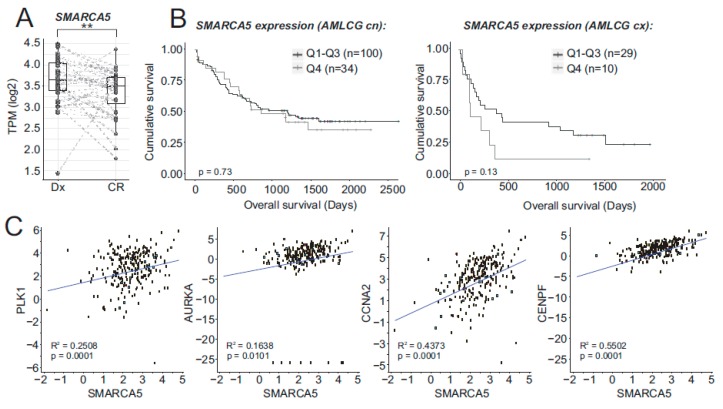
(**A**) SMARCA5 expression of matched AML samples at the time of diagnosis (Dx) and complete remission (CR). Dots represent individual samples; dashed lines connect matched patient samples. Boxes: distribution of the Dx and CR groups; intermediate line = median. Significance was estimated using a paired Wilcoxon test. (**B**) Survival analysis of AML patients divided into quartiles (from low Q1 to high Q4; Q1: 0–25% + Q2: 25–50% + Q3: 50–75% vs. Q4: 75–100%) based on SMARCA5 mRNA levels (cn: cytogenetically normal, cx: cytogenetic abnormalities). (**C**) Correlation of mRNA levels of PLK1, AURKA, CCNA2, CENPF, and SMARCA5 (R^2^ and p-value indicated).

Based on previous evidence documenting SMARCA5 overexpression in small AML patient subset [19], we examined RNAseq data of bone marrow samples from AML patients with recorded overall survival (OS). We confirmed our previous observation [19] that *SMARCA5* levels are significantly elevated at the time of diagnosis and decreased after the patients achieved complete hematologic remission (Figure 1A). We next associated SMARCA5 expression and clinical parameters and (due to genetic AML heterogeneity) followed separately cytogenetically normal (CN) and abnormal (CX) AML patients. Hence, we could observe a trend for decreased OS in the AML patient population with higher SMARCA5 expression and carrying cytogenetic abnormalities (Figure 1B). We also observed that higher *SMARCA5* levels correlated with mRNA expression of proliferation biomarkers such as AURKA, PLK1, CCNA2, CENPF (Figure 1C).

### 2.2. SMARCA5 Deletion Inhibits AML Cell Proliferation

**Figure 2:**
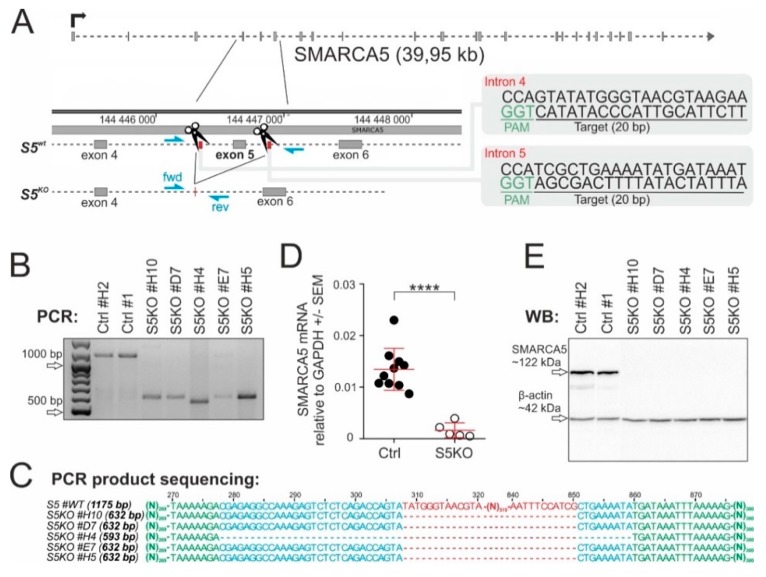
Inactivation of *SMARCA5* gene expression (S5KO) in AML cells. (**A**) Scheme of generating the S5KO using CRISPR/Cas9 technology. The Cas9 nuclease was targeted into two intronic sites (scissors) surrounding exon5 of the *SMARCA5* gene. The sequences of guide RNAs are depicted in gray boxes on the right. Indicated are exons 4-6 (small rectangles) and genotyping primers (blue arrowheads). (**B**) PCR verification of the exon5 deletion in the indicated S5KO clones. (**C**) Analysis of *SMARCA5* gene region following the Cas9 nuclease deletion. PCR products (same as in B) were Sanger-sequenced and aligned with the wt sequence using the Kalign web tool. After sequencing, the precise length of the resultant PCR amplified region was determined (on the left in brackets). (**D**) Quantitative PCR analysis of *SMARCA5* mRNA expression in the S5KO clones (*n* = 5) compared to controls (*n* = 10). Data normalized to the GAPDH mRNA. Student’s *t*-test, *p* < 0.00001 ****. (**E**) Immunoblotting of SMARCA5 expression in CRISPR/Cas9-treated K562 or controls. β-actin controlled the load.

To test requirement of SMARCA5 for AML cell growth, we produced a null allele using CRIPSR/Cas9 genome editing technology (Figure 2A). Targeted was exon5, which codes a portion of evolutionarily conserved ATPase domain and that was previously shown to be a targetable region using the Cre-loxP1 system. Deletion of exon5 results in a frame shift mutation disabling expression of Smarca5 protein in mouse [1]. For the experiments, human K562 cells (AML M6 subtype) were initially utilized as they were previously used for antisense oligonucleotide-mediated transient knockdown of SMARCA5 [2]. K562 cells were transfected by a pair of pX330-mVenus vectors containing sgRNAs complementary to a sequence in the SMARCA5 introns 4 & 5 and the the effect of CRISPR/Cas9-mediated deletion of exon5 was tested by PCR. Analysis of fragments amplified from genomic DNA of FACS-sorted mVenus-positive clonal populations identified 5 clones (#H10, D7, H4, E7, H7) with a single shortened PCR product (~632bp compared to 1175bp in controls) that were homozygously mutated (Figure 2B). Sanger sequencing of PCR products confirmed that clones H10, D7, E7, and H5 contained the same deletion (543bp) and clone H4 an even larger deletion (582bp) within SMARCA5 exon5 (Figure 2C). In addition, quantitative PCR and Western blot analyses of the cellular extracts confirmed that the Cas9-mediated deletion of the SMARCA5 gene resulted in loss of SMARCA5 expression (Figure 2D,E). The resulting subclones had no expression of vector-coded & episomally expressed Cas9 nuclease. In addition, eight predicted off-target candidates (SRGAP2, RNF17, PRG4, GYPA, POLQ, CYB5R4, BCKDHB, NAV2) had no alteration of their sequences. Thus, we managed to effectively delete SMARCA5 in the K562 subclones to create a cellular model for studying how SMARCA5 loss affected AML cell growth.

### 2.3. Smarca5 Deletion Inhibits Proliferation of Myeloblasts and Affects Function of Normal Stem Cells

**Figure 3:**
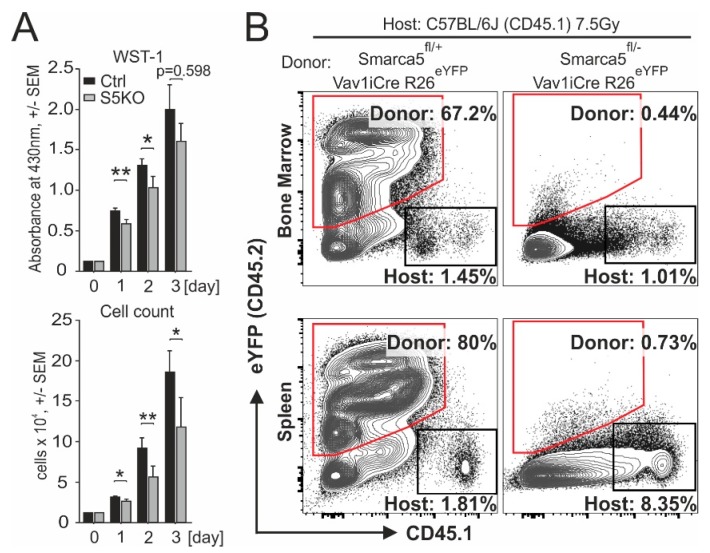
Proliferation of AML and progenitor cells upon *Smarca5* gene deletion. (**A**) Proliferation of S5KO clone #D7 and control cells analyzed by WST-1 assay. Mean ± SEM of formazan absorbance (top) and cell count (bottom) (pentaplicates). Student’s *t*-test, *p* < 0.05 *, *p* < 0.01 **. (**B**) Flow cytometry analysis of donor (CD45.2) and host (CD45.1) derived hematopoietic cells at 14 days following the transplantation of donor fetal liver cells into lethally (7.5 Gy) irradiated host animals. Donor (red trapezoid) and host-derived (black rectangles) bone marrow cells (upper dot plots) and splenocytes (lower dot plots) were distinguished by the expression of yellow fluorescent protein (eYFP) or surface variant of CD45. Control mice: Smarca5^fl/+^ Vav1iCre R26^eYFP^; Smarca5 mutant mice: Smarca5^fl/fl^ Vav1iCre R26^eYFP^. Data are representative of repeated experiments.

To characterize the effect of *SMARCA5* deletion in the AML-S5KO subclones, we monitored their growth in culture by the WST-1 assay correlating the number of metabolically active cells in the 72-hr culture within a 96-well plate. We quantitated the data with a scanning multiwell spectrophotometer (ELISA reader) (Figure 3A, upper panel) and also in parallel counted the viable cells with an automated cell counter (Figure 3A, lower panel). We observed that starting day 1, the S5KO subclones produced less formazan product/s compared to AML ‘control’ cells, indicating that loss of SMARCA5 impaired proliferation of leukemic cells. We also attempted to create S5KO clones from additional AML cell lines. We repeatedly used OCI-M2, NB4, SKM1, MOLM-13, however, despite the fact that these AML cell lines grew normally in tissue culture conditions, the recombined cells by pX330-mVenus vectors followed by the single cell sorting could not produce clones with exon5 deletion. We therefore used the method of serial dilution of transfected cells. This approach, in contrast to the previous approach, produced populations of OCI-M2 and SKM1 cell lines with detectable Cas9-edited *SMARCA5* loci. However, the signals of mutated alleles markedly decreased during long-term cultivation, suggesting that the S5KO cells were overgrown by cells containing at least one intact SMARCA5 allele. Thus, the deletion of the *SMARCA5* gene completely impaired leukemic cell proliferation in most of the AML cell lines, while in K562 cells it was tolerated albeit under markedly lower proliferation activity, which allowed us to study it in more detail. 

AML cell population resembles early hematopoietic progenitors. Thus, as controls to AML cells, we studied early murine blood progenitors. Previously it was shown that Smarca5 loss in mouse partially inhibits differentiation of early Lin^−^Sca-1^+^c-Kit^+^ hematopoietic progenitors [1]. To test whether Smarca5 deletion affects reconstitution of early blood progenitors after transplanting them into normal murine recipients, we utilized the hematopoietic reconstitution assay. We transferred E13.5 mouse fetal liver cells (C57Bl/6J Ly5.2 background) isolated either from control Smarca5^flox/+^ Rosa26^eYFP/+^ Vav1-iCRE or *Smarca5*-deficient (Smarca5^flox/−^ Rosa26^eYFP/+^ Vav1-iCRE) embryos into lethally irradiated adult C57Bl/6J Ly5.1 recipients. Flow cytometric analyses of bone marrow and spleen at several weeks after transplantation revealed that repopulation was detected only in animals transplanted with cells in which the *Smarca5* gene was preserved. Thus, homeostatic expression of Smarca5 is very important for hematopoietic reconstitution (Figure 3B), implicating a possibility that the Smarca5 role in AML cells might also involve a very early leukemia-initiating compartment.

### 2.4. Inactivation of Smarca5 Causes Nuclear Abnormalities and Polyploidy

**Figure 4:**
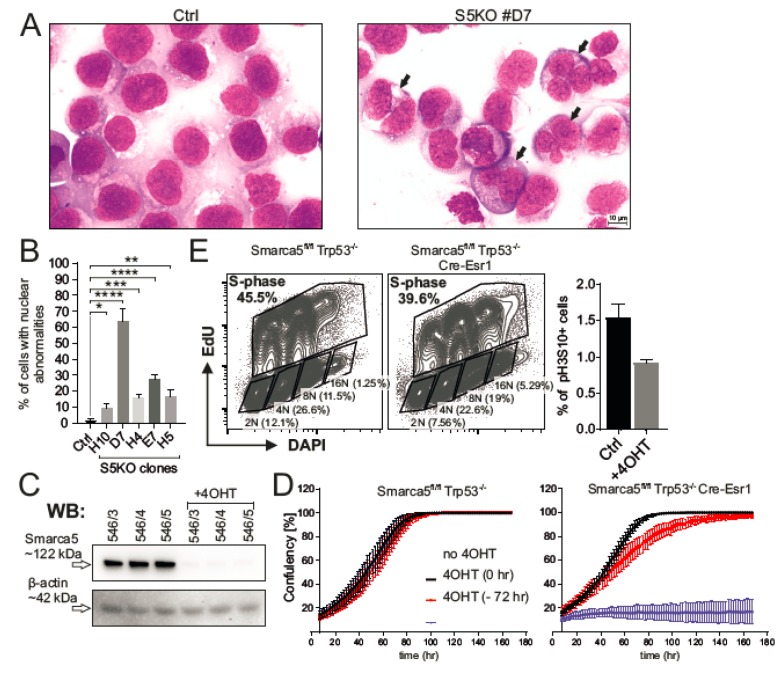
Nuclear abnormalities in S5KO cells. (**A**) Cytology of control (left) and S5KO clone #D7 (right), nuclear abnormalities indicated and shown (**B**) as mean % ± Stdev of control, 400 cells/subclone analyzed. Student’s *t*-test, *p* < 0.05 *, *p* < 0.01 **, *p* < 0.0001 ****. (**C**) Immunoblotting of Smarca5: MEF cell lines (Smarca5^fl/fl^ Cre-Esr1: untreated, 4OHT-treated (100 nM, 6 h exposure, 4 days of culture). β-actin = loading control. (**D**) IncuCyte cell proliferation analysis; control Smarca5^fl/fl^ (upper panel) vs. Smarca5^fl/fl^ Cre-Esr1 (lower panel) MEFs in absence/presence of 4OHT (100 nM, 6 h exposure), or alternatively, 4OHT was added 72 h prior to IncuCyte monitoring (4OHT—72 h). *Y*-axis: mean confluency (%) and ± Stdev of at least 16 different regions of the cultivation plate, *X*-axis: time (h). (**E**) Flow cytometry analysis of control and Smarca5^fl/fl^ Cre-Esr1 MEF population cell cycle progression using EdU/DAPI double staining (upper dot plots). Black rectangles depict all S-phase and non-S-phase cells with different ploidy (2N-16N). Histograms show percentage of phospho-histone H3 (Ser10) positive mitotic events in experimental cell lines. (**D**) and (**E**) represent biological triplicates.

To gain insight into the subcellular structures of the AML S5KO cells, we utilized hematology staining using a standardized May–Grunwald and Giemsa–Romanowski stain procedure. As indicated within Figure 4A, the control AML cells were represented by a uniform layer of myeloblasts with large round nuclei, fine chromatin structure, and prominent nucleoli. Significantly more frequent nuclear abnormalities were observed in the S5KO cells compared to controls. These included nuclear budding, internuclear bridging, karyorrhexis, and multinuclearity seen in 10% to 65% of all analyzed cells (Figure 4B). To study effect/s of S5 depletion in nonhematopoietic cells, we derived mouse embryonic fibroblast (MEF) with Tamoxifen-regulated Cre-recombinase activity (Cre-Esr1) from *Smarca5^fl/fl^ Trp53^−/−^* animals. *Trp53*-mutated MEFs were chosen because of their lower propensity to enter proliferation senescence and because most AML cell lines including K562 have *TP53* gene inactivation [22]. After 6 h incubation with 100 nM 4-hydroxy-tamoxifen (4OHT) and additional 90 h of culture, the MEF cells were depleted from Smarca5 protein (Figure 4C). Decrease of Smarca5 protein level negatively influenced the cell growth and the proliferation defect had already occurred within 40 h from the start of the 4OHT treatment while 4OHT untreated and control Cre-Esr1 lacking cells proliferated normally (Figure 4D). This proliferative defect resembled one observed in AML S5KO clones. The flow cytometry analysis revealed that aberrant proliferation was accompanied by lower proportion of S-progressing and mitotic (pH3S10^+^) cells. In addition, we noted a higher number of cells with polyploid nuclei (Figure 4E) that was concomitant to a decreased proportion of diploid cells upon S5 deficiency in MEFs. Taken together, inactivation of SMARCA5 triggers a cell proliferation blockade and results in nuclear abnormalities of exceedingly cycling leukemic as well as normal hematopoietic cells.

### 2.5. Cytogenetic Abnormalities and Gene Expression Dysregulation in the S5KO AML Cells

**Figure 5:**
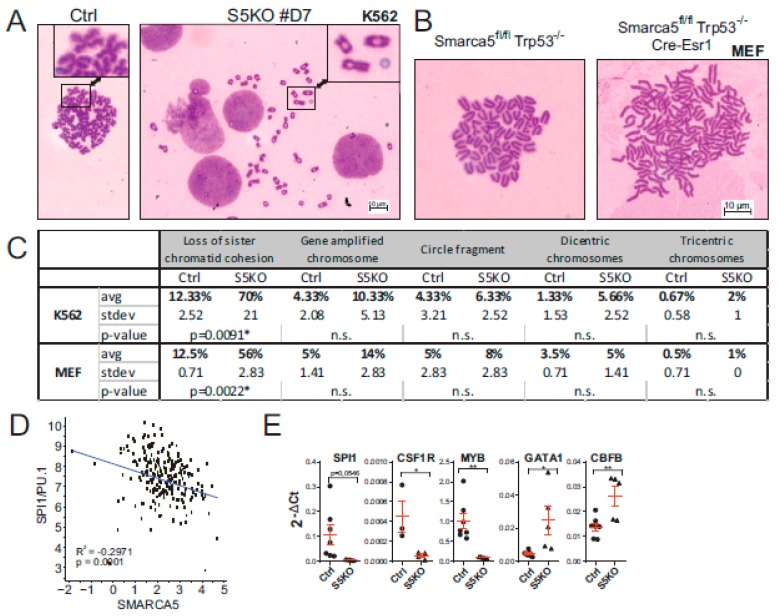
SMARCA5 loss causes karyotypic changes in K562 cells. (**A**,**B**) Mitotic chromosome analysis of S5KO cells vs control K562 cells (clone #D7, (**A**)) or MEF cells (**B**). 1000X magnification. (**C**) Table summarizes all chromosomal aberrations; data from technical triplicates, for each replicate a total of 100 mitotic nuclei were counted. Mean percentage of chromosomal abnormalities with Stdev, Student’s *t*-test, *p* < 0.05 *. (**D**) Computational analysis of correlations between expression of SPI1/PU.1 and SMARCA5 in samples of adult AML patient samples; for details, see Materials and Methods section. (**E**) Quantitative PCR analysis of SPI1, CSF1R, MYB, GATA1, and CBFB mRNAs expression in the S5KO clones (*n* = 5) compared to controls (*n* = 7). Data were normalized to the GAPDH mRNA. Student’s *t*-test, *p* < 0.05 *, *p* < 0.001 **.

As pointed out in the Introduction section, SMARCA5 protein was previously shown to load cohesin complex onto human chromosomes [23]. As the canonical role of cohesin is the sister chromatid cohesion, we next analyzed the structures of mitotic chromosomes in the AML S5KO cells on metaphase spreads. The analysis of the S5KO subclone D7 consistently showed (Figure 5A) that among other chromosomal abnormalities, the cohesion defects were by far the most frequent involving premature chromatid separation and loss of cohesion. Compared to the controls that contained only 12%, the S5KO mitotic cells displayed defects in chromatin cohesion in 70% of all cases. Similarly, the defects of chromatid cohesion were seen also in MEF cell-derived mitotic chromosome spreads (Figure 5B,C). These data suggest that SMARCA5 inhibition affects cohesin function in general. 

In order to better understand the cooperative nature of SMARCA5 and its interacting partners in AML, we correlated their expression using RNAseq data in AML patients. Hence, significant association exists between the expression pattern of SMARCA5 and BAZ proteins (BAZ1A, BAZ1B, BAZ2A, BAZ2B) as well as the members of the CTCF/cohesin complex across human AML samples. This implicates, albeit indirectly, a role of SMARCA5 in CTCF/cohesin function in AML that also coincides with karyotype abnormalities imposed by a SMARCA5 loss.

We recently showed that SMARCA5 (together with the CTCF/cohesin complex) represses PU.1-mediated myeloid differentiation [7] and similarly, we noted that SMARCA5 regulates GATA1-mediated erythropoiesis [1]. We therefore next decided to analyze the levels of SPI1/PU.1 and GATA-1 transcripts with respect to SMARCA5. As expected, transcriptomic data from AML Cooperative Group München (Figure 5D) showed an inverse correlation between SPI1/PU.1 and SMARCA5 expression in AML patient samples. To further assess the role of SMARCA5 in regulation of the hematopoietic transcription program, we determined the expression of a set of selected mRNAs upon the genetic ablation of the *SMARCA5* gene in K562 cells. Compared with previously published data documenting an inverse relationship between SMARCA5 and hematopoietic transcription factors PU.1 or GATA-1, we observed that upon SMARCA5 deletion in K562 cells the level of SPI1/PU.1 and some of its targets (CSF1R) became downregulated while other transcription factors (GATA1, CBFB) were upregulated. The dysregulation of mRNA pattern of SMARCA5 targets upon SMARCA5 deletion can be attributed to the heterogeneity of the AML cell lines and also possibly to multiple genetic/cytogenetic abnormalities imposed by the *SMARCA5* loss.

## 3. Discussion

We herein studied how ISWI ATPase SMARCA5/SNF2H controls in AML the proliferation and gene expression of myeloblasts as SMARCA5 appeared to be an interesting target for anti-AML therapy. Our previous work demonstrated a pattern of SMARCA5 upregulation at AML diagnosis followed by its normalization upon achieving the hematologic remission. Importantly, additional work has not identified recurrent mutations of SMARCA5 in AML or any malignant disease (so far analyzed by next-generation sequencing-based techniques). For example, for the *SMARCA5* gene, only 186 variants with an amino acid residue substitution exist in nearly ~20 thousand oncologic patients (<1%). There also exist infrequently the variants in ISWI-interacting BAZ proteins detected in cancer, however, the significance of these variants remains also unknown. Importantly, among the AML-associated variants, only the SMARCA5-interacting proteins, CTCF and members of the cohesin complex, were shown consistently mutated in AML [24]. Based on this, we expected SMARCA5 indispensability for AML proliferation and its levels possibly reflecting the proliferative nature of AML cells. Indeed, the RNAseq analysis of a large set of AML patients confirmed that AML cells overexpressed SMARCA5 and its levels correlated with many ISWI-complex members including also cohesin complex, and finally, that the proliferative nature of AML cells marked by upregulation of SMARCA5 was supported by a trend in shorter OS albeit only in those AML patients that were marked by cytogenetic aberrations (see Figure 1). 

Upon targeting of the *SMARCA5* gene in AML cell lines with a CRISPR/Cas9-mediated deletion strategy, we could observe that AML cells lacking *SMARCA5* markedly slowed the proliferation rate and became dysplastic with multiple karyotypic abnormalities. Inhibiting SMARCA5 to achieve suppression of AML growth may be thus a very efficient strategy as AML cells that are likely addicted to SMARCA5 in order to overcome various chromatin obstacles such as complex karyotype or also polyploidy often seen during progression of AML. Other data further implicated that SMARCA5 is very important also at the stem cell level to regulate their innate function: to repopulate the progeny. Indeed (as shown by Figure 3), repopulation activities were greatly reduced in normal hematopoietic stem cells in which the *Smarca5* gene was genetically deleted. Our observation, however, does not rule out the possibility of SMARCA5 being an AML target as i) the AML cells are highly proliferating compared to their normal counterparts, and ii) SMARCA5 being expressed in stem cells implicates that antiSMARCA5 therapy would preferentially target the leukemia stem and progenitor cells. 

While SMARCA5 expression represents a potential target for AML therapy, it may also serve as a factor of therapeutic resistance in AML. It is likely that additional factors will be involved in modulating therapy efficacy using SMARCA5 inhibitors in the future. As the Smarca5 loss was sensed in a mouse model by a) increased p53 levels and b) associated with DNA damage response (DDR), and c) activation of the p53 targets [1], very likely the tumor cells with DDR sensing defect would have a higher propensity to tolerate SMARCA5 level downregulation. This notion is supported by our other study demonstrating that proliferation defect imposed by Smarca5 deficiency can be partly restored with concomitant *Trp53* deletion in murine thymocytes [3].

Our herein presented data indicate that AML growth is dependent on the expression of chromatin remodeling protein SMARCA5 that is a known partner of AML-associated targets: cohesin complex and CTCF [23]. Data presented in Figure 4 and Figure 5 implicate that proliferation inhibition upon *SMARCA5* targeting is at least in part caused by karyotype abnormalities, especially cohesion defects, and possibly also by a putative replication defect due to defective chromatin compaction as well as dysregulation of gene expression pattern of the key hematopoietic lineage restricted transcription factors. Interestingly, the nuclear changes after S5 deletion such as polyploidy were also described in other cell lines of hematologic origin [1,3] but not as a result of *Smarca5* deletion of developing brain or eye lens [4,5]. Similar evidence was noted upon experimental manipulation with cohesin complex members; for example, the nonsense mutations in STAG2 (generated in the THP1 AML cell line) led to defects in sister chromatid cohesion and induced anaphase defects, which resulted in proliferation blockade [25]. Important connections between replication and cohesion have been established in the HeLa tumor cells, in which the interfering with replication affected chromatid cohesion and caused a defect in mitotic progression [26]. Others suggested that cohesion defects depend on a functional mitotic spindle checkpoint in regulating mitotic progression [27]. It seems that the strategy of inhibiting SMARCA5 in AML to block leukemogenesis becomes even more vital as shown recently using inhibitors of SMARCA5 (ED2-AD101) that target the HELICc-DExx domain to release the terminal AML cells into differentiation while sparing normal hematopoiesis in preclinical animal models [28]. Our work also suggests that upon inhibiting SMARCA5-mediated proliferation of AML cells, we also can face the problem of inhibiting proliferation of normal cells. Further work in this respect on experimental animals is under way. An additional strategy to inhibit AML cell growth specifically could be to target the *SMARCA5* exon5 in AML cells by CRISPR/Cas9 as evidenced by the herein presented data. Data from global CRISPR/Cas9 screen identified that SMARCA5 targeting was very efficient and caused cell growth inhibition in several additional AML cell lines (OCI-AML2, OCI-AML3) and also in lymphoma and carcinoma cell lines [21]. Together, our as well as others’ data demonstrate that SMARCA5 is a valuable epigenetic target suitable for inhibitor discovery projects and subsequent validation in MDS/AML and potentially also in other types of cancer.

## 4. Materials and Methods

### 4.1. CRISPR Vector Design

pX330-Venus (kindly provided by Dr. Bjoern Schuster) produces CRISPR/Cas9 enzyme that cleaves at a specific location based on sequence guide sgRNA defined target sequences in SMARCA5 intron4 (5′-TTCTTACGTTACCCATATACTGG-3′) and SMARCA5 intron5 (5′-ATTTATCATATTTTCAGCGATGG-3′). CRISPR/Cas9 enzyme is also fused with fluorescent protein mVenus, that enables selection of successfully transfected clones by FACS sorting. The DNA sequences for the sgRNA SMARCA5 intron4 and sgRNA SMARCA5 intron5 were synthesized by Sigma-Aldrich as four oligonucleotides with modifications at position 1 (to encode a Guanine due to the transcription initiation requirement of the human U6 promoter). These two pairs of complementary oligos were mixed together, boiled at 95 °C for 10 min, and allowed to cool down to RT to hybridize. Double-stranded oligos also designed with complementary BbsI overhangs on 3′ and 5′ ends were ligated into BbsI linearized pX330-Venus vector using T4 Ligase enzyme (Thermo Fisher Scientific, Waltham, MA, USA). Ligation mixtures were transformed into Subcloning Efficiency DH5α Competent Cells (Invitrogen, Carlsbad, CA, USA) following the manufacturer’s protocol. pX330-Venus sgRNA hSMARCA5 intron4 and pX330-Venus sgRNA hSMARCA5 intron5 were isolated and purified by GenElute HP Plasmid Midiprep kit (Sigma-Aldrich, St. Louis, MO, USA) and correct oligo insertion verified by Sanger sequencing. 

### 4.2. Cell Lines

K562 cells (ATCC, Manassas, Virginia, USA) were cultured in 90% Iscove’s Modified Dulbecco’s Medium supplemented with 10% fetal bovine serum and 1% penicillin/streptomycin. NB4, SKM-1, and MOLM-13 were cultured in 90% RPMI-1640 medium (Sigma-Aldrich), OCI-M2 in 80% Iscove’s Modified Dulbecco’s medium (Biosera, Kansas City, MO, USA) at 37 °C and 5% CO2. The media were supplemented with 10-20% fetal bovine serum (Biosera) and 1% penicillin/streptomycin (Biosera). Cell lines were purchased from DSMZ. Both pX330-Venus sgRNA SMARCA5 intron4 (1 μg) and px330-Venus sgRNA SMARCA5 intron5 (1 μg) were transfected into 2.5 × 10^6^ K562 cells using Amaxa Cell Line Nucleofector kit (Lonza, Basel, Switzerland) and 1 × 10^6^ K562 cells using Neon Transfection System 10 μL Kit (Invitrogen). Cells were cultivated for 48 h, Venus-positive cells sorted on BD FACS Aria Fusion and divided to form single cell clones on 96-well plates. DNA from growing clones was used as a template for PCR with the following primers: forward 5′-GAGATGGAGGGCTACACTGTG-3′and reverse 5′-GACATTCCCAAAGTCATCTAGCAG-3′. The resulting amplification produced 1175 bp fragment from wild-type and approximately 632 bp long fragment from CRISPR/Cas9 edited allele of the SMARCA5 gene. Cell smears (0.5–1 × 10^5^ cells) were fixed with methanol and stained with May–Grünwald solution (mixed 1:1 with distilled water, Penta, Limassol, Cyprus) for 5 min and Giemsa–Romanowski solution (mixed 1:13 with distilled water, Penta) for 12 min. Cell Proliferation Reagent WST-1 (Roche, Basel, Switzerland) was used following manufacturer’s protocol starting from day 0 with seeding 0.5 × 104 cell/100 μL/well in triplicates and continued by daily measurement of absorbance at 430 nm on microplate reader Infinite 200 PRO (Tecan, Männedorf, Switzerland). Cells were simultaneously counted by Luna Automated Cell Counter (Logos Biosystems, Dongan, South Korea). 

### 4.3. AML Patients and Statistics

RNA-Seq data sets from AML patient samples were previously described including the informed consent and ethical issues [29,30,31]. Reads were mapped with STAR aligner version 2.7.2d using GRCh37 reference and annotation version 32 from GENCODE (www.gencodegenes.org). Reads were counted using FeatureCounts version 1.6.5, normalized to transcripts per million (TPM) and log2 transformed. Log-rank test was performed in survival analysis, Wilcoxon test was used to assess differences in gene expression.

### 4.4. Real-Time qPCR

Total RNA from wild-type (*n* = 10) and knockout (*n* = 5) K562 clones was isolated by TRIzol Reagent (Invitrogen) and reverse-transcribed by High Capacity cDNA Reverse Transcription kit (Thermo Fisher Scientific). Quantitative PCR was run in triplicates on LightCycler 480 (Roche) using LightCycler 480 SYBR Green I Master (Roche) and specific primers for human SMARCA5 (forward primer 5′-AACTTACTATCCGTTGGCGATT-3′, reverse primer 5′-GGTTGCTTTGGAGCTTTCTG-3′) and GADPH (forward 5′-AGCCACATCGCTCAGACAC-3′, reverse primer 5′-GCCCAATACGACCAAATCC-3′) gene. Ct values served for fold-change calculation using 2-ΔΔCt equation. Student’s *t*-test was used for statistical analysis.

### 4.5. Western Blot

Wild-type and S5KO K562 clones (1 × 10E7) were lysed in RIPA Buffer (Sigma-Aldrich) supplemented with protease and phosphatase inhibitors (Roche). Denatured cell lysates were run on 1 mm thick 10% SDS-PAGE gel (40 μg/lane) in Mini-Protean Electrophoresis system (Bio-Rad, Hercules, CA, USA) and semi-dry-blotted onto PVDF membrane (Bio-Rad) using Trans-Blot Turbo transfer system (Bio-Rad). PVDF membrane was blocked for 1 h in 5% nonfat milk in 1x TBS/0.1% Tween-20 and incubated with primary antibodies: Snf2h/ISWI (Bethyl Laboratories Inc., #A301-017A-1, Montgomery, TX, USA) and β-actin (Santa Cruz Biotechnology, #sc-1616-R, Dallas, Texas, USA) overnight at 4 °C. Horseradish peroxidase-conjugated secondary antibodies (anti-rabbit, anti-goat) visualized bands using Pierce ECL Western Blotting substrate (Thermo Fisher Scientific). 

### 4.6. Cytogenetics

Standard cytogenetic methods published previously [10,11] were used for preparation of slides, with few modifications. Briefly, the K562 cells were synchronized with colcemid (10 µl/mL) at 37 °C and hypotonized in 0.075 M KCl for 20 min. The cells were then fixed in three changes of cold Carnoy’s fixative (ethanol: glacial acetic acid, 3:1) and dropped onto a slide inclined at an angle of 45 degrees from a height. The chromosomal preparations were air-dried overnight and stained using 5% Giemsa blue solution (Sigma-Aldrich) prepared in standard Sorenson buffer. Preparations were inspected under a light microscope BX43 (Olympus, Sony, Shinjuku, Japan) with microscope camera Infinity 2-2 (Lumenera, Ottawa, ON, Canada). Selected plates were photographed under a 100x immersion oil objective using software QuickPHOTO CAMERA 3.1 (Olympus).

### 4.7. Hematopoietic Reconstitution 

For hematopoietic reconstitution experiments, 2.5 × 10^6^ fetal liver cells isolated from E13.5 control (Smarca5^fl/+^ Rosa26^eYFP/+^ Vav1-iCRE) and Smarca5-deficient (Smarca5^fl/−^ Rosa26^eYFP/+^ Vav1-iCRE) with C57Bl/6J Ly5.2 background were transplanted into lethally irradiated (7.5 Gy) adult (8 weeks) C57Bl/6J Ly5.1 recipients. After 12 days, the recipients were euthanized, and their bone marrow and spleen were tested for the presence of donor-derived eYFP+ hematopoietic cells using flow cytometry. The antibody panel included CD45.1, CD45.2, c-Kit, Sca1, and lineage cocktail (CD3, B220, Mac-1, Gr-1, Ter119).

### 4.8. Analysis of S5KO MEF Cells

S5KO MEF cells (*n* = 3) were isolated from E14.5 embryos, in which the *Smarca5* gene contained the LoxP1 sites upstream and downstream of exon5 and also expressed Cre Recombinase-Estrogen receptor fusion protein that translocated into the nucleus upon addition of 4OHT into the cultures for 6 h. Deletion of *Smarca5*-exon5 represents a null allele [2]. Production of stable MEF cells was enabled by concurrent deletion of *Tp53* gene [32]. Gene targeting of the Smarca5^flox/flox^ Cre-Esr1 cells upon 4OHT addition was confirmed by previously published detection methods [2]. Analysis of cell proliferation of MEFs was determined by IncuCyte (Sartorius, Göttingen, Germany) that enables analysis in 96 wells under real-time continuous visualization and monitoring.

## Abbreviations

SMARCA5	SWI/SNF-Related, Matrix-Associated, Actin-Dependent Regulator of Chromatin, Subfamily A, Member 5
SNF2H	Sucrose Nonfermenting Protein 2 Homolog
AML	Acute Myeloid Leukemia
CRISPR	Clustered Regularly Interspaced Short Palindromic Repeats

## References

[1] Kokavec J., Zikmund T., Savvulidi F., Kulvait V., Edelmann W., Skoultchi A.I., Stopka T. (2017). The ISWI ATPase Smarca5 (Snf2h) Is Required for Proliferation and Differentiation of Hematopoietic Stem and Progenitor Cells. Stem Cells.

[2] Stopka T., Skoultchi A.I. (2003). The ISWI ATPase Snf2h is required for early mouse development. Proc. Natl. Acad. Sci. USA.

[3] Zikmund T., Kokavec J., Turkova T., Savvulidi F., Paszekova H., Vodenkova S., Sedlacek R., Skoultchi A.I., Stopka T. (2019). ISWI ATPase Smarca5 Regulates Differentiation of Thymocytes Undergoing beta-Selection. J. Immunol..

[4] Alvarez-Saavedra M., De Repentigny Y., Lagali P.S., Raghu Ram E.V., Yan K., Hashem E., Ivanochko D., Huh M.S., Yang D., Mears A.J. (2014). Snf2h-mediated chromatin organization and histone H1 dynamics govern cerebellar morphogenesis and neural maturation. Nat. Commun..

[5] He S., Limi S., McGreal R.S., Xie Q., Brennan L.A., Kantorow W.L., Kokavec J., Majumdar R., Hou H., Edelmann W. (2016). Chromatin remodeling enzyme Snf2h regulates embryonic lens differentiation and denucleation. Development.

[6] Barisic D., Stadler M.B., Iurlaro M., Schubeler D. (2019). Mammalian ISWI and SWI/SNF selectively mediate binding of distinct transcription factors. Nature.

[7] Dluhosova M., Curik N., Vargova J., Jonasova A., Zikmund T., Stopka T. (2014). Epigenetic control of SPI1 gene by CTCF and ISWI ATPase SMARCA5. PLoS ONE.

[8] Morris S.A., Baek S., Sung M.H., John S., Wiench M., Johnson T.A., Schiltz R.L., Hager G.L. (2014). Overlapping chromatin-remodeling systems collaborate genome wide at dynamic chromatin transitions. Nat. Struct. Mol. Biol..

[9] Goodwin L.R., Picketts D.J. (2018). The role of ISWI chromatin remodeling complexes in brain development and neurodevelopmental disorders. Mol. Cell Neurosci..

[10] Erdel F., Rippe K. (2011). Chromatin remodelling in mammalian cells by ISWI-type complexes—Where, when and why?. FEBS J..

[11] Kadoch C., Crabtree G.R. (2015). Mammalian SWI/SNF chromatin remodeling complexes and cancer: Mechanistic insights gained from human genomics. Sci. Adv..

[12] Garraway L.A., Lander E.S. (2013). Lessons from the cancer genome. Cell.

[13] Dutta A., Sardiu M., Gogol M., Gilmore J., Zhang D., Florens L., Abmayr S.M., Washburn M.P., Workman J.L. (2017). Composition and Function of Mutant Swi/Snf Complexes. Cell Rep..

[14] Gigek C.O., Lisboa L.C., Leal M.F., Silva P.N., Lima E.M., Khayat A.S., Assumpcao P.P., Burbano R.R., Smith Mde A. (2011). SMARCA5 methylation and expression in gastric cancer. Cancer Investig..

[15] Reis S.T., Timoszczuk L.S., Pontes-Junior J., Viana N., Silva I.A., Dip N., Srougi M., Leite K.R. (2013). The role of micro RNAs let7c, 100 and 218 expression and their target RAS, C-MYC, BUB1, RB, SMARCA5, LAMB3 and Ki-67 in prostate cancer. Clinics.

[16] Sheu J.J., Choi J.H., Yildiz I., Tsai F.J., Shaul Y., Wang T.L., Shih Ie M. (2008). The roles of human sucrose nonfermenting protein 2 homologue in the tumor-promoting functions of Rsf-1. Cancer Res..

[17] Jin Q., Mao X., Li B., Guan S., Yao F., Jin F. (2015). Overexpression of SMARCA5 correlates with cell proliferation and migration in breast cancer. Tumour. Biol..

[18] Zhao X.C., An P., Wu X.Y., Zhang L.M., Long B., Tian Y., Chi X.Y., Tong D.Y. (2016). Overexpression of hSNF2H in glioma promotes cell proliferation, invasion, and chemoresistance through its interaction with Rsf-1. Tumour. Biol..

[19] Stopka T., Zakova D., Fuchs O., Kubrova O., Blafkova J., Jelinek J., Necas E., Zivny J. (2000). Chromatin remodeling gene SMARCA5 is dysregulated in primitive hematopoietic cells of acute leukemia. Leukemia.

[20] Rosenbauer F., Wagner K., Kutok J.L., Iwasaki H., Le Beau M.M., Okuno Y., Akashi K., Fiering S., Tenen D.G. (2004). Acute myeloid leukemia induced by graded reduction of a lineage-specific transcription factor, PU.1. Nat. Genet..

[21] Behan F.M., Iorio F., Picco G., Goncalves E., Beaver C.M., Migliardi G., Santos R., Rao Y., Sassi F., Pinnelli M. (2019). Prioritization of cancer therapeutic targets using CRISPR-Cas9 screens. Nature.

[22] Law J.C., Ritke M.K., Yalowich J.C., Leder G.H., Ferrell R.E. (1993). Mutational inactivation of the p53 gene in the human erythroid leukemic K562 cell line. Leuk. Res..

[23] Hakimi M.A., Bochar D.A., Schmiesing J.A., Dong Y., Barak O.G., Speicher D.W., Yokomori K., Shiekhattar R. (2002). A chromatin remodelling complex that loads cohesin onto human chromosomes. Nature.

[24] Welch J.S., Ley T.J., Link D.C., Miller C.A., Larson D.E., Koboldt D.C., Wartman L.D., Lamprecht T.L., Liu F., Xia J. (2012). The origin and evolution of mutations in acute myeloid leukemia. Cell.

[25] Kim J.-S., He X., Orr B., Wutz G., Hill V., Peters J.-M., Compton D.A., Waldman T. (2016). Intact Cohesion, Anaphase, and Chromosome Segregation in Human Cells Harboring Tumor-Derived Mutations in STAG2. PLOS Genet..

[26] Leman A.R., Noguchi C., Lee C.Y., Noguchi E. (2010). Human Timeless and Tipin stabilize replication forks and facilitate sister-chromatid cohesion. J. Cell Sci..

[27] De Lange J., Faramarz A., Oostra A.B., de Menezes R.X., van der Meulen I.H., Rooimans M.A., Rockx D.A., Brakenhoff R.H., van Beusechem V.W., King R.W. (2015). Defective sister chromatid cohesion is synthetically lethal with impaired APC/C function. Nat. Commun..

[28] Kishtagari A.N., Jarman C., Tiwari A.D., Phillips J.G., Schuerger C., Jha B.K., Saunthararajah Y. (2018). A First-in-Class Inhibitor of ISWI-Mediated (ATP-Dependent) Transcription Repression Releases Terminal-Differentiation in AML Cells While Sparing Normal Hematopoiesis. Blood.

[29] Herold T., Jurinovic V., Batcha A.M.N., Bamopoulos S.A., Rothenberg-Thurley M., Ksienzyk B., Hartmann L., Greif P.A., Phillippou-Massier J., Krebs S. (2018). A 29-gene and cytogenetic score for the prediction of resistance to induction treatment in acute myeloid leukemia. Haematologica.

[30] Stief S.M., Hanneforth A.L., Weser S., Mattes R., Carlet M., Liu W.H., Bartoschek M.D., Dominguez Moreno H., Oettle M., Kempf J. (2020). Loss of KDM6A confers drug resistance in acute myeloid leukemia. Leukemia.

[31] Batcha A.M.N., Bamopoulos S.A., Kerbs P., Kumar A., Jurinovic V., Rothenberg-Thurley M., Ksienzyk B., Philippou-Massier J., Krebs S., Blum H. (2019). Allelic Imbalance of Recurrently Mutated Genes in Acute Myeloid Leukaemia. Sci. Rep..

[32] Jacks T., Remington L., Williams B.O., Schmitt E.M., Halachmi S., Bronson R.T., Weinberg R.A. (1994). Tumor spectrum analysis in p53-mutant mice. Curr. Biol..

